# Acquisition and Analysis of Facial Electromyographic Signals for Emotion Recognition

**DOI:** 10.3390/s24154785

**Published:** 2024-07-24

**Authors:** Marcin Kołodziej, Andrzej Majkowski, Marcin Jurczak

**Affiliations:** Faculty of Electrical Engineering, Warsaw University of Technology, Pl. Politechniki 1, 00-661 Warsaw, Poland; marcin.kolodziej@pw.edu.pl (M.K.); marcin.jurczak.dokt@pw.edu.pl (M.J.)

**Keywords:** electromyography, EMG, signal analysis, emotion recognition, expression recognition, facial analysis

## Abstract

The objective of the article is to recognize users’ emotions by classifying facial electromyographic (EMG) signals. A biomedical signal amplifier, equipped with eight active electrodes positioned in accordance with the Facial Action Coding System, was used to record the EMG signals. These signals were registered during a procedure where users acted out various emotions: joy, sadness, surprise, disgust, anger, fear, and neutral. Recordings were made for 16 users. The mean power of the EMG signals formed the feature set. We utilized these features to train and evaluate various classifiers. In the subject-dependent model, the average classification accuracies were 96.3% for KNN, 94.9% for SVM with a linear kernel, 94.6% for SVM with a cubic kernel, and 93.8% for LDA. In the subject-independent model, the classification results varied depending on the tested user, ranging from 91.4% to 48.6% for the KNN classifier, with an average accuracy of 67.5%. The SVM with a cubic kernel performed slightly worse, achieving an average accuracy of 59.1%, followed by the SVM with a linear kernel at 53.9%, and the LDA classifier at 41.2%. Additionally, the study identified the most effective electrodes for distinguishing between pairs of emotions.

## 1. Introduction

Emotion recognition plays a key role and is extremely important from the perspective of the development of affective computing [1]. One of the main reasons is to improve human-computer interaction [2]. Modern technologies and user interfaces are becoming increasingly advanced, but they often lack a human dimension [3]. Emotion recognition allows computers to better understand and respond to users’ emotions, enabling more personalized and intuitive interactions that consider the mood, preferences, and needs of the user. This can lead to higher user satisfaction and better adaptation of interfaces to individual requirements. Emotion recognition enables computer systems to gather information about the emotional state of users [4]. It can also be used in healthcare, including in the diagnosis and monitoring of patients’ emotional states [5]. Assistance in detecting symptoms of depression, anxiety, or other disorders can enable faster and more effective interventions, benefiting patients, therapists, and the entire healthcare system [5].

Many attempts have been made to effectively recognize emotions. Most commonly, these tasks utilize techniques of facial image processing and the analysis of electrophysiological signals. Facial image analysis is one of the most popular techniques used in recognizing user emotions, as facial expressions are a natural way of expressing emotions [6,7]. A drawback of facial image processing and analysis techniques is that facial expressions must be noticeable on the person’s face, which is usually possible only when experiencing significant emotions [8]. Therefore, ongoing research is focused on methods of detecting emotions using other measurement techniques.

The analysis of electrophysiological signals is another important technique used to recognize user emotions. In this context, the most commonly used methods are electroencephalography (EEG), electromyography (EMG), and electrodermal activity (EDA) [9,10]. EEG records the brain’s electrical activity, enabling inferences about a user’s emotional state [11]. EMG analyzes muscle activity, allowing for the detection of muscle tension associated with various emotions, such as anger or joy [12]. EDA measures the electrical activity of the skin, which is related to the level of emotional arousal. The analysis of electrophysiological signals provides objective and direct information about a user’s emotional state.

Electromyography serves as an advanced tool in studies of the facial expression of emotions. With its precision, ability to detect micro-expressions, and objectivity of results, EMG enables a deeper understanding of the processes of expressing emotions [13,14]. Discoveries from EMG research are significant for the field of psychology and other disciplines interested in human emotions and non-verbal communication [15,16,17,18,19,20]. Facial electromyography is more precise and reliable in analyzing muscle activity associated with emotional expression than emotion detection using a camera [21,22]. Facial electromyography allows for the direct measurement of electrical muscle activity, enabling accurate monitoring of muscle operation during emotional expression. Unlike cameras, which only observe facial expressions, EMG provides more objective data about muscle activity. Facial electromyography is more effective in detecting quick and subtle muscle movements that may be overlooked or invisible to a camera [23]. It provides detailed information about the degree of activation of individual muscles, allowing for a more precise determination of specific emotions expressed on the face.

In [24], an EMG dataset acquired under an emotional environment by Augsburg University is analyzed. The dataset consists of 96 EMG signals representing four emotions. Four features, including root mean square, variance, mean absolute value, and integrated EMG, are calculated. These parameters are provided to three different classifiers: the Elman neural network (ENN), backpropagation neural network (BPNN), and nonlinear autoregressive exogenous network (NARX), for emotion classification. In [25], a study is conducted to enable machines to recognize emotions from facial electromyogram signals. This includes a compilation of the group’s attempts to recognize basic facial expressions, namely happy, angry, and sad, using EMG signals from facial muscles. The group extracted features from three EMG signals from the faces of two human subjects, one male and one female, and analyzed these features to serve as feature templates. In the study [26], a portable wireless transmission system for multichannel acquisition of EMG signals around the face was proposed. The multichannel approach allows this system to simultaneously detect muscle activity in 16 regions. To recognize these movements, three classifiers were used: random forest, support vector machine (SVM), and a backpropagation neural network. The paper [27] surveys and analyzes methods, strengths, and challenges of using biosignals for detecting emotional states. It proposes a framework that combines facial expression detection, using EMG, with saccade detection using electrooculography (EOG), to classify four basic emotions: neutral, sad, happy, and angry. In [28], it is demonstrated that signals from distal electrode locations on areas of low facial mobility exhibit strong amplitude and correlate with signals captured in traditional positions atop facial muscles. The study reports on the choice of electrode position and the successful identification of facial expressions using computational methods. It also proposes a wearable interface device that can detect facial bioelectrical signals distally in a continuous manner while being unobtrusive to the user. This proposed device can be worn on the side of the face to capture signals that are considered a mixture of facial electromyographic signals and other bioelectrical signals. In [16], an analysis of 30 scientific papers dedicated to recording, processing, and classifying signals from facial surface EMG for emotion detection and human-computer communication is presented. Signals were recorded using one to eight EMG channels. For classification, EMG signal fragments ranging from 64 ms to 1042 ms were utilized. Various methods were employed in the feature extraction process from the electromyographic signal, with the most common techniques utilizing the signal’s energy. The extracted features were subsequently used for classification. Diverse methods served as classifiers, including linear discriminant analysis (LDA), thresholding, support vector machine (SVM), Gaussian classifier, and k-nearest neighbors (KNN) classifier. In the study [29], a device was constructed to detect subtle spontaneous expressions related to smiling using several electrodes. Eight voluntary participants took part in the research, and the results indicate high effectiveness in detecting smiles.

### Aim of the Article

In scientific literature on facial electromyography in emotion recognition, we found no comprehensive study evaluating the feasibility of recognizing emotions using electrodes aligned with the Facial Action Coding System (FACS). The integration of EMG electrodes with FACS enables precise tracking of facial muscle activity in the context of specific emotional expressions, allowing for a more reliable and detailed analysis of facial expressions.

This article aims to develop an effective method for recognizing emotions through EMG signals recorded from the face. We used a biomedical signal amplifier with eight active electrodes to record these signals, choosing electrode locations based on publications and the Facial Action Coding System [30]. EMG signals were recorded during a procedure where users acted out emotions such as joy, sadness, surprise, disgust, anger, fear, and a neutral state, portraying these emotions subtly and naturally. Signals from 16 users were recorded and divided into segments for analysis. We utilized the average power in the time domain as a signal feature. We tested k-nearest neighbors (KNN), support vector machines (SVM), and linear discriminant analysis (LDA) classifiers, presenting results for both user-dependent and user-independent approaches. Additionally, the study included an analysis of the recorded EMG signals and the identification of the most effective electrodes for classifying pairs of emotions.

Possible applications of EMG signals, despite the inconvenience of signal collection to some extent, primarily include medicine and rehabilitation. For example, EMG-based emotion recognition systems can be used to monitor the emotional state of patients with neurological disorders, allowing for more precise therapy adjustments. Additionally, in clinical settings, such systems can support the rehabilitation of stroke patients by helping to assess their progress through the analysis of facial muscle activity.

In practice, muscle activity can be more conveniently read using a camera, which is less invasive and more comfortable for users. However, EMG technology can offer greater precision and accuracy in analyzing subtle changes in muscle activity that may be invisible to cameras. Furthermore, EMG signals can provide real-time data, which is crucial in applications requiring quick response, such as human-machine interfaces or vehicle safety systems. Despite certain inconveniences, the precise data provided by EMG can significantly enhance the effectiveness and reliability of many applications.

## 2. Materials and Methods

### 2.1. Experiment Setup and Signal Acquisition

**Figure 1:**
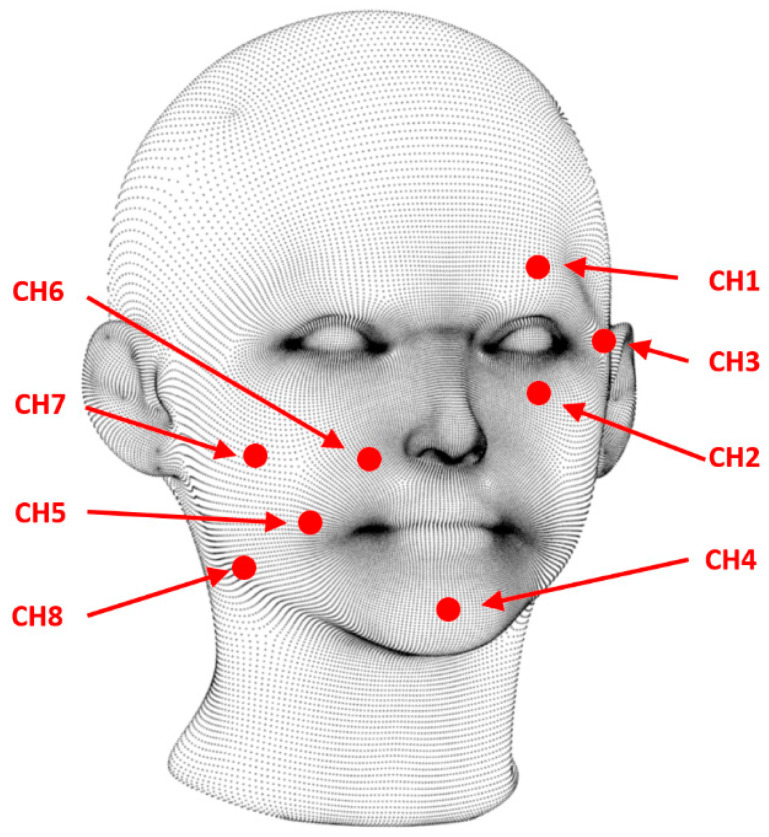
Placement of EMG electrodes on the face.

For the research, we created a database of EMG signals registered while subjects acted out various emotions. These signals were recorded using a g.Tec g.USBAmp 2.0 24 bit bioelectrical signal amplifier and g.LADYBIRD series active electrodes. The recording setup included eight measuring electrodes, a reference electrode (Ref), and a ground electrode (Gnd) to equalize the amplifier potential. We placed the EMG electrodes over key facial muscles, selecting these muscles in accordance with Ekman and Friesen’s Facial Action Coding System (FACS) [31,32]. The analysis resulted in the selection of six electrodes directly associated with muscles activated during emotional experiences. Signals from two electrodes, C4 (located on the chin) and C8 (on the jaw), were added to obtain a better spatial representation of the potentials across the entire facial area. Figure 1 illustrates the locations of the EMG electrodes (CH1–CH8). The electrodes were positioned asymmetrically. We chose not to increase the number of electrodes on the face due to concerns that a larger number might hinder users’ ability to perform facial expressions. Table 1 presents a compilation of emotions, the facial muscles associated with these emotions, the expressions performed, and the corresponding electrodes.

Inducing emotions in experiment participants is a challenging task because emotional responses vary significantly due to numerous factors. One of the challenges is that individuals do not all react in the same way to the same emotional stimuli. Variations in life experiences, personality, and cultural context mean that what elicits intense emotions in one person may be met with indifference or have a slightly different impact on another. Sixteen men (S01–S16), comprising students and employees of the Warsaw University of Technology, aged 20 to 57, participated in the experiments. The average age of the participants was 32.3 ± 13.7 years. Each participant provided consent for the research. They were seated in an armchair at a desk equipped with a monitor displaying brief information about their tasks. Additionally, participants received brief training explaining the experiment’s procedure.

During registration, participants were instructed to perform specific emotions (joy, sadness, surprise, disgust, anger, and fear) within 30 s or to relax and do nothing (neutral state). Throughout these 30 s, participants were to spontaneously perform brief facial expressions corresponding to the presented emotions. They were asked to express each emotion briefly and gently, followed by a one-second pause. Participants in the study were allowed to express emotions freely, without specific training on how to enact each emotion. They were asked to convey the emotions through facial expressions in a manner they felt genuinely reflected their reactions. The authenticity of these emotional expressions was not verified, nor was there an attempt to standardize the expression of emotions among participants. We recognize that, in this case, facial expressions may not reflect the participants’ actual emotions.

The signals recorded while presenting the fixation cross served as the “baseline”. The sequence of tasks was randomized, with a 10-s interval between each emotion. Participants were advised to pause for at least 2 s before executing another activity. The registration period lasted 6 min and 30 s. Each registration session was conducted on a different day in the morning under similar conditions.

In the next step, specific segments of the EMG signals recorded during the experience of emotions were selected. For each emotion and the neutral state, ten fragments of 1/4-s EMG signal were chosen. An experienced EMG technician selected these signal fragments. The window length of 1/4 s was selected based on observations of the EMG signals for all users. This duration represents a compromise, allowing for the observation of changes related to muscle activity without classifying the EMG signal based on longer segments, which could include different emotions and facial expressions occurring sequentially. Consequently, a database was created containing 10 records of EMG signals for each participant (S01–S16), recorded during the performance of individual emotions and the neutral state. The recorded database has been made available on the Internet.

Table 2 provides basic information about the database created for the research. This database encompasses data from eight EMG channels, collected from sixteen participants who expressed six different emotions (joy, surprise, fear, anger, sadness, disgust) and a neutral state. For each emotion and participant, 10 signal fragments were collected, resulting in a total of 160 fragments per emotion for all participants. Overall, the database gathered 960 examples of emotions and 160 examples of a neutral state, amounting to 1120 examples in total.

### 2.2. Signal Preprocessing and Feature Extraction

The OpenViBE 3.4.0 software was utilized for designing the presentation scenario and acquiring signals. The signals were recorded at a high sampling frequency of 4800 Hz. During recording, a high-pass filter of 0.1 Hz and a low-pass filter of 1000 Hz were implemented. Additionally, a 50 Hz Butterworth 4th-order notch filter was used. Observation of the spectrum of recorded EMG signals revealed disturbances caused by the transmission of harmonic components of the power grid (100 Hz, 150 Hz, 200 Hz,..., 1000 Hz). To address this, the recorded signals were filtered using Butterworth notch filters of the 4th order for each of these frequencies. The EMG signals were not subjected to any other preprocessing methods.

**Figure 2:**
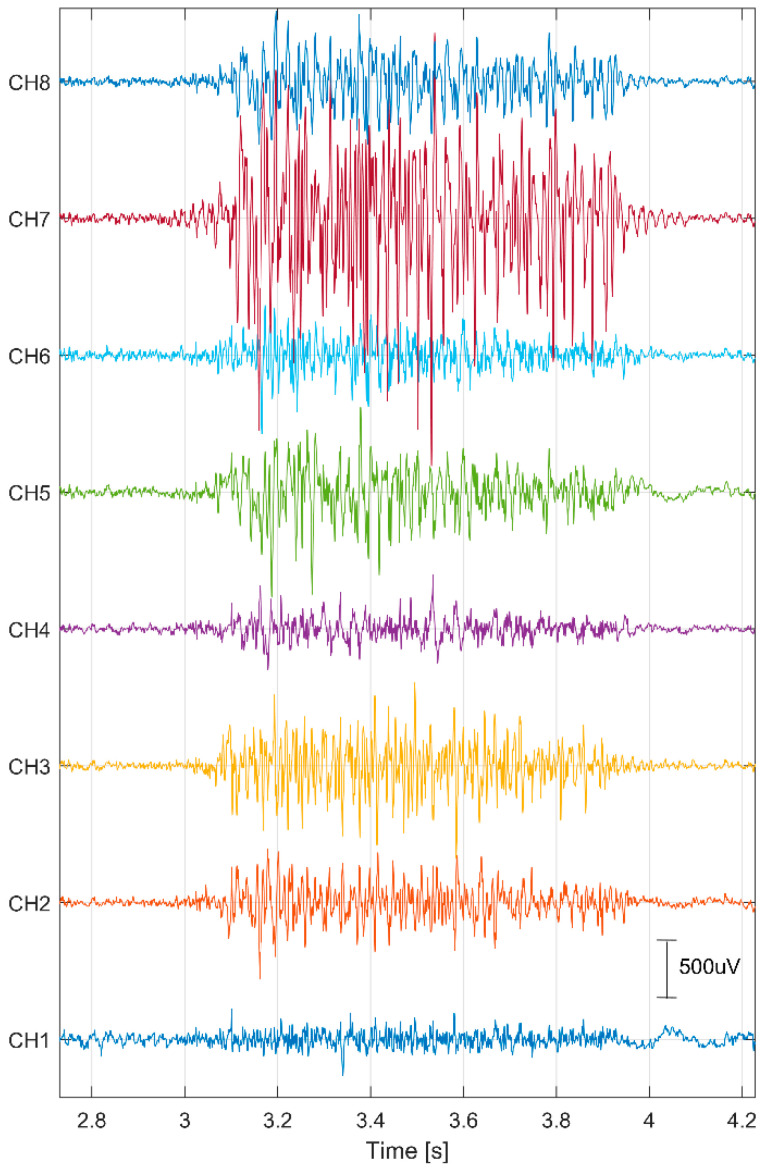
Recording of EMG signals while expressing joy.

Figure 2 displays a segment of the recorded EMG signals while expressing joy. The highest amplitude values were observed on electrode CH7, positioned on the cheek of the face. In our study, we decided to use a computationally simple and easy-to-interpret feature of the EMG signal, namely, the average power in the time domain. The average power in the time domain was calculated for each 1/4-s of the signal and for each channel [33]:

(1) $$P_{x}=\frac{1}{N}\sum_{n=0}^{N-1}{\left|x\left[n\right]\right|}^{2},$$

where *N* is the number of samples in the window, and *x*[*n*] is the value of the *n*-th sample. The calculated values have a unit of µV^2^. Average power in the time domain is a crucial feature in the analysis of EMG signals [34]. It measures the overall muscle activity within a specific timeframe.

We chose signal power for emotion recognition based on EMG signals for several reasons. First, signal power is a simple and effective indicator of muscle activity, correlating well with various emotional states. It provides a stable measure despite noise and is widely used in biomedical signal analysis for its reliability and intuitiveness. Although advanced techniques like wavelet analysis exist, they often do not significantly improve accuracy. Additionally, the variability in emotional expression can impact classification accuracy more than feature extraction complexity. Thus, signal power simplifies data processing, enhances model generalization, and is easier to implement in real-world conditions.

### 2.3. Classification of Emotions

The classification problem involves assigning objects to predefined classes based on their features or attributes. In addressing this problem, various classifiers can be employed. Common classifiers include k-nearest neighbors (KNN), support vector machines (SVM), and linear discriminant analysis (LDA) [35,36]. Practically, experimenting with different algorithms and fine-tuning their parameters can yield optimal classification results. Therefore, we tested several classifiers.

Measuring the effectiveness of emotion recognition is crucial in research on emotion recognition systems. Classification accuracy [9] is the most commonly used metric for this purpose. It evaluates how effectively a model or algorithm classifies emotions based on available test data. A widely used method for assessing accuracy is 10-fold cross-validation (10-CV). In the 10-CV, each of the 10 data subsets are used both for training and testing. This is particularly vital when data is expensive or difficult to acquire. A confusion matrix is also a valuable tool for assessing the effectiveness of emotion recognition. It is a table that displays the number of correctly and incorrectly classified cases for each class. This matrix offers detailed insights into which classes were accurately recognized by the model and which were not.

## 3. Results and Discussion

### 3.1. Analysis of Recorded EMG Signals

At the outset of our research, we conducted a detailed analysis of the power of facial muscle activity during emotional expressions. Analyzing the power distributions on individual electrodes helps us understand the mechanisms behind emotional expression, providing more in-depth insights into the physiological manifestation of each emotion. Additionally, this analysis allows us to verify the accuracy of the recorded EMG signals and check their consistency with the widely accepted knowledge about emotions and the activation of specific facial muscles.

**Figure 3:**
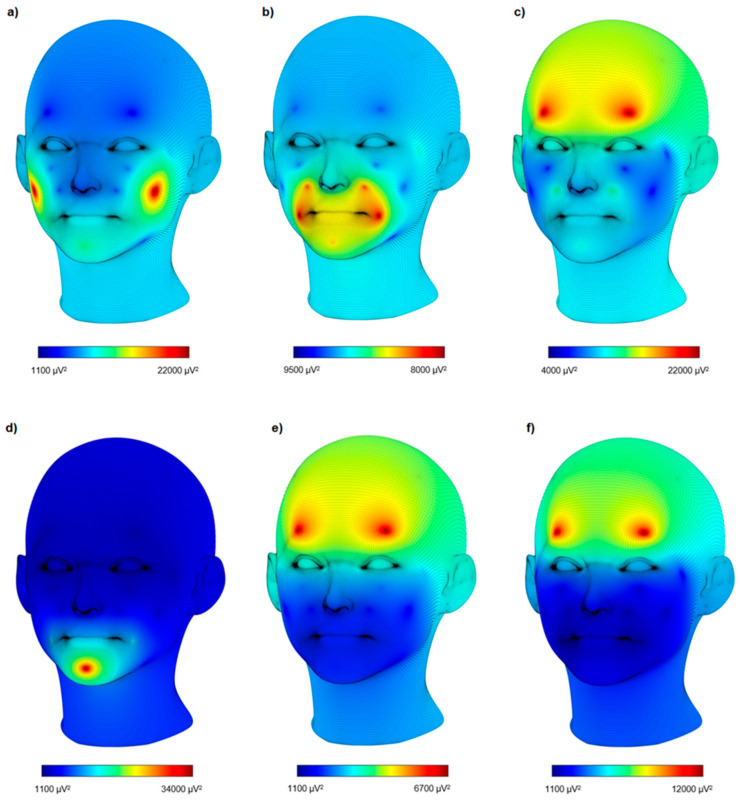
Distribution of signal power while expressing (**a**) joy, (**b**) disgust, (**c**) anger, (**d**) sadness, (**e**) fear, and (**f**) surprise.

An analysis of electromyographic data obtained from eight electrodes labeled CH1-CH8 was conducted. The average power calculated in the time domain served as a measure to describe muscle activity. Table 3 includes the results of the EMG analysis for different emotions (neutral, joy, sadness, surprise, disgust, anger, fear) and statistics for each electrode (CH1-CH8), encompassing both the mean and standard deviation (std). The neutral state is characterized by low muscle activity, with electrode CH4 showing higher mean activity compared to other electrodes. For joy, electrode CH7 is pivotal, exhibiting the highest muscle activity, although electrode CH4 also shows significant activity. The signal power for electrodes CH1, CH2, CH3, and CH7 is higher, indicating activity in the Orbicularis oculi and Zygomaticus major muscles. In the case of surprise, electrode CH1 shows significant activity associated with eyebrow raising. For fear, electrode CH1 displays significant activity, but the response of CH2 is less pronounced compared to other emotions. For anger, electrodes CH1, CH2, and CH3 all demonstrate an increase in signal power. Sadness shows greater activity in CH1 and CH5. Lastly, for disgust, electrode CH6 demonstrates a significant increase in response, corresponding to the lifting of the upper lip and wrinkling of the nose. These results are consistent with findings based on Ekman and Friesen’s Facial Action Coding System [31], where facial expressions are divided into action units representing individual facial movements. Figure 3 shows the distribution of signal power while expressing joy, disgust, anger, sadness, fear, and surprise.

**Figure 4:**
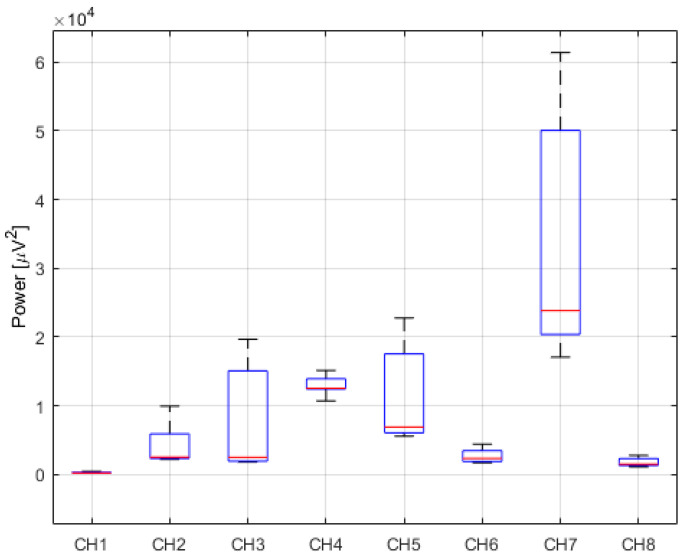
Distribution of signal power while expressing joy.

Figure 4, in turn, presents the analysis results in the form of boxplots, illustrating the distribution of data regarding muscle activity while expressing joy. The graph shows the differences in EMG signal amplitude recorded by electrodes CH1 to CH8.

### 3.2. Emotion Recognition on Data from All Experiment Participants

In the next stage of our research, we aimed to explore the potential for emotion recognition using data from all experiment participants. To evaluate the effectiveness and reliability of the proposed classification model, we employed a 10-fold cross-validation approach. The samples for 10-CV were randomly assigned, ensuring that data from each participant were evenly distributed across the folds. This method allowed for a more objective evaluation of the classification efficiency, reducing the risk of overfitting the model and providing insights into its generalization under different conditions. The EMG signal feature was the average power calculated in the time domain for each channel (CH1 to CH8). All computed features from eight electrodes were utilized for the classification. We utilized all 1120 examples of 6 emotions and the neutral state recorded for all 16 individuals for training.

For the SVM classifier with a cubic kernel, an accuracy of 0.893 was achieved. The KNN classifier attained an accuracy of 0.909, the SVM with a linear kernel reached 0.678, and the LDA scored 0.524. Given that the classification encompassed seven distinct classes of emotions, these results are considered satisfactory. It is noteworthy that better outcomes were observed with classifiers that consider nearest neighbors (KNN) and those accounting for non-linear relationships between features. Table 4 displays the confusion matrix obtained during the 10-fold cross-validation (10-CV) test using the KNN classifier. Analyzing the presented confusion matrix for emotion recognition, it is evident that the classifier generally performed well in correctly assigning examples to their respective classes. In the case of the neutral state, a majority of examples (148) were accurately classified, although some misclassifications occurred, primarily with the fear (6 examples), anger (1 example), and disgust (5 examples). Joy was recognized effectively, with 160 examples correctly classified. Similarly, surprise was accurately identified with 157 correct classifications, albeit with minor errors in other categories. Fear and anger appeared to be more challenging to distinguish, with some examples incorrectly classified between these emotions, as well as with disgust. The classification of sadness encountered some errors, mainly involving the neutral state and fear. Disgust, while generally well classified (136 examples), also experienced some misclassifications, particularly with fear (16 examples), neutral state (4 examples), anger (2 examples), and sadness (2 examples).

Therefore, while the classifier achieved good overall results, certain emotions such as fear, anger, and disgust proved more difficult to distinctly differentiate.

### 3.3. Electrode Selection for Distinguishing Pairs of Emotions

Continuing our research on the accuracy of emotion classification, we opted to examine more closely which electrodes are most effective in distinguishing between individual pairs of emotions and the neutral state. This investigation will enable us to select the most useful electrodes for classification purposes. Additionally, it will facilitate a more detailed examination of the distinguishability of specific emotions from one another. Table 5 presents the results of classification accuracy using the SVM classifier with a cubic kernel for individual pairs of emotions and electrodes CH1-CH8.

The results presented are based on the 10-fold cross-validation (10-CV) test. The highest classification accuracy was attained using electrodes CH2 and CH8 for distinguishing between the neutral state and joy, achieving an accuracy of 1.00. Other electrodes also showed high effectiveness. In the classification of the neutral state versus surprise, electrodes CH2, CH4, CH5, CH6, and CH8 reached accuracy ranging from 0.93 to 0.98. For differentiating the neutral state from fear, electrodes CH2, CH3, CH5, CH6, CH7, and CH8 proved most effective, with accuracy ranging from 0.90 to 0.93. In classifying the neutral state as opposed to anger, electrodes CH2, CH6, CH7, and CH8 achieved accuracies ranging from 0.91 to 1.00. For the classification of the neutral state compared to sadness, electrodes CH2, CH3, CH6, and CH7 reached accuracies from 0.93 to 0.99. In differentiating the neutral state from disgust, electrodes CH1, CH7, and CH8 achieved accuracies of 0.79, 0.90, and 0.95, respectively. When classifying joy versus fear, electrodes CH2, CH6, CH7, and CH8 showed accuracies of 0.91, 0.87, 0.99, and 0.78, respectively. In the classification of joy versus disgust, electrodes CH1, CH2, CH7, and CH8 attained accuracies of 0.74, 0.82, 0.99, and 0.93, respectively.

Overall, the analysis revealed that certain electrodes, specifically CH2, CH6, CH7, and CH8, are particularly effective in distinguishing specific pairs of emotions. The accuracy in many instances exceeded 0.9, underscoring the high effectiveness of these electrodes in emotion classification.

### 3.4. Emotion Recognition in a Subject-Dependent Approach

In the subsequent phase of our research, we investigated the feasibility of classifying emotions using a subject-dependent approach, where the system is trained and tested for a specific user. The average power in the time domain was employed as an EMG signal feature for training and testing classifiers. In this approach, only examples recorded for a single individual were used. Table 6 presents the results of classifying emotions and the neutral state for 16 different individuals, employing various algorithms such as k-nearest neighbors (KNN), support vector machines with a linear kernel (SVM_linear), support vector machines with a cubic kernel (SVM_cubic), and linear discriminant analysis (LDA).

The metric presented is the classification accuracy obtained from the 10-fold cross-validation test. The average classification accuracy indicates that the LDA classifier achieves the highest average effectiveness, with a score of 0.960, highlighting its potential usefulness in emotion classification. However, it is important to note that both the KNN and SVM with a linear kernel also attained good results, with average scores of 0.941 and 0.918, respectively. The low standard deviations imply that the results are stable and consistent. However, the variation in results for individual subjects suggests the influence of individual characteristics on the effectiveness of the algorithm. For certain individuals, notably better (Subject 12) or worse (Subject 6) results were observed.

As an illustration, Table 7 presents a confusion matrix for the SVM classifier with a linear kernel for user S03, where the classification accuracy achieved is 0.971. The confusion matrix illustrates the algorithm’s effectiveness in classifying different emotions. Emotions such as surprise, fear, anger, and disgust seem to be recognized effectively, as all instances of these emotions were correctly classified.

However, the algorithm faces some challenges in distinguishing between joy and sadness, as evidenced by several classification errors. The confusion matrix offers valuable insights into the algorithm’s performance and highlights areas requiring improvement. The results obtained are superior compared to the approach in which data from all experiment participants were used, indicating that models based on user-specific data might provide enhanced accuracy in emotion recognition.

### 3.5. Emotion Recognition in a Subject-Independent Approach

In the next stage of our research, we explored the feasibility of classifying emotions using a subject-independent approach. This means that the training and testing data came from different users, making the test more challenging and realistic. In the experiment, the data were divided so that the system was trained using data from 15 users, while testing was conducted on data from a single remaining user. This process was then repeated for each of the 16 users, with each user being selected as the test subject in turn. The aim of this approach was to determine whether the system could be trained to recognize emotions based on data from multiple users and then correctly identify emotions in a new user who was not part of the training. This is particularly important in the context of practical applications for emotion recognition systems, where it is expected that the system will be effective for new users.

We tested four different classifiers: k-nearest neighbors (KNN), support vector machine (SVM) with a linear kernel, support vector machine with a cubic kernel, and linear discriminant analysis (LDA). Each of these classifiers was tasked with recognizing seven different emotion classes: neutral state, joy, sadness, surprise, disgust, anger, and fear. The classification accuracy results for individual users in the subject-independent approach are presented in Table 8.

The presented results clearly show that the accuracy of emotion classification varies for each user and across different classifiers. The KNN classifier achieved high accuracy for most users, particularly for users 3, 7, and 16, with accuracies of 0.871, 0.829, and 0.914, respectively. However, for user 10, the accuracy was only 0.486, indicating difficulties in classifying emotions for this specific user. The SVM with a linear kernel showed variable accuracy, with the lowest accuracy for user 2 (0.300) and the highest for user 16 (0.971). This classifier generally had lower accuracy compared to the others, suggesting that a linear separation of the feature space is insufficient for effective emotion classification in this dataset. The SVM with a cubic kernel demonstrated better performance than its linear counterpart. User 6 achieved the highest accuracy of 0.771, and user 3 also had good accuracy at 0.643. However, the accuracy for user 10 was low (0.329), similar to the KNN results, suggesting that certain emotions may be difficult to classify for this user regardless of the classifier used. The LDA classifier had the weakest performance among all classifiers. The highest accuracy was achieved for user 16 (0.943) and user 7 (0.800). For most users, the accuracy was significantly lower; for example, user 1 had an accuracy of only 0.157. These results indicate that LDA may not be a suitable classifier for this task, likely due to the linear separation of classes.

There is significant variability in classification accuracy among different users for each classifier. This indicates individual differences in EMG signals, which impact the effectiveness of classification. KNN and SVM with a cubic kernel appear to be more effective compared to SVM with a linear kernel and LDA, suggesting that nonlinear classification methods may be better suited for this task. Table 9 presents a sample confusion matrix for user S01 and the KNN classifier in the subject-independent approach. The neutral state was recognized best, as all 10 instances were correctly classified without any errors. Similarly, fear was also well-recognized, with 10 correct classifications and no errors. The worst-recognized were surprise and disgust. For surprise, only 3 out of 10 instances were correctly classified, with the remaining instances misclassified as neutral state (2 instances), fear (1 instance), and disgust (4 instances). Disgust had only 1 correct classification out of 10, with the remaining instances misclassified as neutral state (3 instances), fear (4 instances), and sadness (2 instances). In summary, the model best recognized the neutral state and fear, as all instances of these classes were correctly classified. The model performed worst with surprise and disgust, where the majority of instances were misclassified as other emotions.

The results indicate that subject-independent classification performs better than random chance, suggesting some consistency in EMG signals among users. However, higher classification accuracy is expected for practical applications. Therefore, we attempted to classify pairs of emotions using a subject-independent approach (Table 10).

We used four classifiers: SVM with a linear kernel (SVM_linear), SVM with a cubic kernel (SVM_cubic), LDA, and KNN. The SVM with a linear kernel achieved the highest accuracy for differentiating the neutral state from joy (0.963) and joy from fear (0.953), while the lowest accuracy was for disgust from anger (0.484) and surprise from fear (0.572). The SVM with a cubic kernel obtained the highest accuracy for differentiating the neutral state from joy (0.984) and joy from sadness (0.978), and the lowest for distinguishing surprise from fear (0.441) and disgust from anger (0.547). The LDA classifier achieved the highest accuracy for distinguishing neutral state from disgust (0.875) and sadness from surprise (0.853), while the lowest accuracy was for distinguishing disgust from anger (0.503) and surprise from fear (0.544). The KNN classifier showed the highest accuracy for differentiating the neutral state from surprise (0.988) and the neutral state from joy (0.978), while the lowest accuracy was for distinguishing surprise from fear (0.619) and surprise from anger (0.659).

The best-recognized emotion pairs are neutral-joy, neutral-surprise, and joy-fear, for which the SVM with a cubic kernel and KNN classifiers achieved very high accuracies. The worst-recognized emotion pairs are disgust-anger and surprise-fear, where all classifiers had significantly lower accuracies. Overall, the nonlinear classifiers (SVM_cubic and KNN) performed better than the linear classifiers (SVM_linear and LDA).

Our study does not account for the variability in the positioning of EMG electrodes on the same individual from day to day. However, our research included multiple users, and despite our best efforts, the electrodes were applied in slightly different positions each time. Additionally, the electrodes, skin, and gel used for attachment affected signal amplification differently. Users also have varying skin impedances and properties, introducing additional variability. Due to these differences, our study, while not conducted as a typical cross-day validation, captured some of these dependencies through the subject-independent approach. Testing on different individuals allowed us to account for the impact of electrode positioning variability and individual skin characteristics, making our models potentially more robust to these factors in real-world applications.

### 3.6. Comparing the Results with Other Studies

Comparing the results of studies focused on emotion recognition using facial EMG signals requires consideration of multiple experimental variables. The specificity of these variables, including the number of EMG electrodes used, the number of study participants, the range and types of recognized emotions, as well as differences in experimental procedures, makes a direct comparison of the obtained results challenging.

Table 11 presents a synthesized compilation of results from seven studies, highlighting the diversity of approaches. We have also provided a summary of dataset’s characteristics, including the number of electrodes and the number of users. This summary helps to better understand the specifics of our dataset and compares it with other available datasets. The results from our subject-dependent approach demonstrate good outcomes, particularly with the use of the k-nearest neighbors classifier, where we achieved a classification accuracy of 0.963. This result is promising and significantly exceeds the average performance of other methods presented in Table 11. In the subject-independent model, the classification results varied depending on the tested user, ranging from 91.4% to 48.6% for the KNN classifier, with an average accuracy of 67.5%. The SVM with a cubic kernel performed slightly worse, achieving an average accuracy of 59.1%, followed by the SVM with a linear kernel at 53.9%, and the LDA classifier at 41.2%.

Each study presented employs slightly different methods for feature extraction and classification. Our research demonstrates that the feature extraction method related to the mean power in the time domain, calculated for a quarter-second time window, is sufficient and offers straightforward and clear interpretation. The chosen classification method can influence the obtained results; however, with a larger number of recorded examples, these variations may amount to only a few percentage points. Regarding the number of participants, our study involved 16 individuals. Expanding the study to include a larger number of participants could provide additional statistical power and enhance the generalizability of our findings.

## 4. Conclusions

In summary, our study, which utilized eight EMG electrodes placed on the face according to the Facial Action Coding System (FACS), allows for effective differentiation of emotions. The application of a simple feature extraction method from the signal, that is mean power in the time domain, enables efficient emotion recognition. The high level of classification accuracy achieved in both subject-dependent and all-participant data approaches suggests that EMG can be effective in practical applications. However, the subject-independent approach did not yield satisfactory results for all participants. Nonetheless, satisfactory classification accuracies were achieved for pairs of emotions. Future research should involve a larger and more diverse group of participants.

## Figures and Tables

**Table 1:** Summary of emotions, facial muscles, and used electrodes.

Emotion	Facial Muscle	Movement	Electrode
Joy	Orbicularis oculi	Eye closure	CH1, CH2, CH3
	Zygomaticus major	Joy	CH7
Surprise	Frontalis	Brow raise	CH1
	Levator palpebrae superioris	Eye widen	CH1, CH2
Fear	Corrugator supercili	Brow furrow	CH1
	Levator palpebrae superioris	Eye widen	CH1, CH2
Anger	Corrugator supercili	Brow furrow	CH1
	Levator palpebrae superioris	Eye widen	CH1, CH2
	Orbicularis oculi	Closing eyelids	CH1, CH2, CH3
Sadness	Frontalis	Brow raise	CH1
	Corrugator supercili	Brow furrow	CH1
	Depressor anguli oris	Lip corner depressor	CH5
Disgust	Levator labii superioris	Upper lip raise	CH6
	Levator labil superioris alaeque nasi	Upper lip raise and nose wrinkle	CH6

**Table 2:** Created database.

Parameter	Value
Number of participants in the study	16 participants
Number of recorded channels	8 EMG channels
Recorded emotions	6 emotions (joy, surprise, fear, anger, sadness, disgust) end 1 neutral state
Number of examples per user for one emotion	10 fragments of signals
Number of examples for all users for one emotion	160 fragments of signals
Number of examples for all users for all emotions	960 examples of emotions and 160 examples of neutral state
Total number of examples	1120 examples

**Table 3:** Statistics of Signal Power [µv^2^] Recorded During Facial Expressions.

Emotion	Statistics	CH1	CH2	CH3	CH4	CH5	CH6	CH7	CH8
Neutral	mean	43.1	14.2	15.1	413.0	14.9	15.8	16.6	16.8
	std	8.8	4.0	3.7	45.4	2.4	3.9	3.4	2.8
Joy	mean	228.0	4046.5	6547.5	12,808.7	10,785.1	2623.0	32,401.9	1740.3
	std	89.8	2747.2	7082.5	1301.0	7116.2	932.6	17,432.8	595.1
Sadness	mean	565.1	790.2	386.9	47,340.5	14,393.9	1504.7	1257.5	599.8
	std	171.2	116.7	64.8	13,183.6	6930.7	185.0	396.4	168.9
Surprise	mean	16,739.7	208.7	178.4	1461.6	246.5	237.2	220.0	254.3
	std	12,045.3	106.5	92.1	997.7	125.1	114.6	106.6	129.5
Disgust	mean	1806.2	1841.5	4231.2	6234.5	10,158.6	8052.7	1439.7	706.4
	std	473.1	361.8	1326.6	1439.6	2605.3	1761.0	443.4	120.6
Anger	mean	29,746.1	4101.8	4453.1	12,128.7	11,137.2	15,476.6	3427.1	7507.6
	std	44,657.3	734.8	2117.1	5056.8	2971.5	3270.7	1381.7	5417.9
Fear	mean	9185.2	1525.1	2123.7	1089.0	1263.7	639.4	143.2	110.3
	std	3426.4	225.3	333.1	383.5	252.0	198.0	23.8	9.8

**Table 4:** Confusion Matrix for All Users—KNN.

		Predicted Class						
		Neutral State	Joy	Surprise	Fear	Anger	Sadness	Disgust
True class	Neutral state	148			6	1		5
	Joy		160					
	Surprise	2		157	1			1
	Fear	7			130	1	1	21
	Anger	3			3	143	8	3
	Sadness	4		1	2	5	144	4
	Disgust	4			16	2	2	136

**Table 5:** Accuracy of Classification for Distinguishing Pairs of Emotions Using Individual Electrodes.

Emotion Pair	CH1	CH2	CH3	CH4	CH5	CH6	CH7	CH8
neutral-joy	0.39	1.00	0.98	0.94	0.95	0.99	0.99	1.00
neutral-surprise	0.45	0.96	0.90	0.97	0.98	0.95	0.80	0.93
neutral-fear	0.60	0.93	0.90	0.43	0.92	0.93	0.91	0.90
neutral-anger	0.73	0.99	0.87	0.34	0.78	1.00	0.97	0.91
neutral-sadness	0.60	0.99	0.98	0.30	0.45	0.93	0.96	0.67
neutral-disgust	0.79	0.70	0.60	0.81	0.73	0.77	0.90	0.95
joy-surprise	0.45	0.54	0.71	0.45	0.44	0.44	0.96	0.46
joy-fear	0.63	0.91	0.12	0.63	0.31	0.87	0.99	0.78
joy-anger	0.51	0.42	0.40	0.44	0.51	0.64	0.62	0.38
joy-sadness	0.56	0.53	0.50	0.50	0.58	0.52	0.80	0.50
joy-disgust	0.74	0.82	0.51	0.66	0.54	0.63	0.99	0.93
surprise-fear	0.54	0.47	0.41	0.51	0.30	0.70	0.35	0.39
surprise-anger	0.36	0.48	0.27	0.25	0.50	0.54	0.44	0.50
surprise-sadness	0.58	0.47	0.39	0.65	0.62	0.45	0.52	0.43
surprise-disgust	0.66	0.61	0.41	0.52	0.51	0.50	0.33	0.41
fear-anger	0.48	0.68	0.35	0.46	0.45	0.78	0.78	0.77
fear-sadness	0.52	0.66	0.41	0.43	0.42	0.86	0.45	0.52
fear-disgust	0.51	0.45	0.50	0.47	0.46	0.54	0.53	0.48
anger-sadness	0.47	0.48	0.43	0.50	0.51	0.54	0.55	0.46
anger-disgust	0.35	0.65	0.55	0.49	0.51	0.56	0.42	0.28
sadness-disgust	0.46	0.56	0.48	0.63	0.53	0.57	0.51	0.58

**Table 6:** Accuracy of Subject-Dependent Classification.

Subject	KNN	SVM_Linear	SVM_Cubic	LDA	Mean
1	0.971	0.829	0.900	0.829	0.882
2	0.900	0.900	0.886	0.943	0.907
3	0.986	0.971	1.000	0.857	0.954
4	0.986	0.971	0.986	0.929	0.968
5	0.929	0.886	0.943	0.914	0.918
6	0.671	0.829	0.829	0.657	0.746
7	0.929	0.929	1.000	0.929	0.946
8	0.914	0.929	0.971	0.886	0.925
9	0.957	0.943	0.957	0.929	0.946
10	0.986	0.871	0.971	0.971	0.950
11	0.986	0.986	1.000	0.957	0.982
12	1.000	0.971	0.986	0.929	0.971
13	0.943	0.886	0.971	0.900	0.925
14	0.986	0.886	1.000	0.943	0.954
15	0.957	0.986	0.986	0.957	0.971
16	0.957	0.914	0.971	0.929	0.943
mean	0.941	0.918	0.960	0.904	
std	0.075	0.050	0.047	0.073	

**Table 7:** Confusion Matrix for User S03.

		Predicted Class						
		Neutral State	Joy	Surprise	Fear	Anger	Sadness	Disgust
True class	Neutral state	10	0	0	0	0	0	0
	Joy	0	9	0	0	1	0	0
	Surprise	0	0	10	0	0	0	0
	Fear	0	0	0	10	0	0	0
	Anger	0	0	0	0	10	0	0
	Sadness	0	0	0	0	1	9	0
	Disgust	0	0	0	0	0	0	10

**Table 8:** Accuracy of Subject-Independent Classification.

Subject	KNN	SVM_Linear	SVM_Cubic	LDA
1	0.614	0.357	0.529	0.157
2	0.643	0.300	0.514	0.229
3	0.871	0.357	0.643	0.243
4	0.571	0.371	0.614	0.143
5	0.557	0.557	0.557	0.371
6	0.586	0.657	0.771	0.457
7	0.829	0.714	0.843	0.800
8	0.700	0.514	0.500	0.657
9	0.671	0.543	0.586	0.329
10	0.486	0.457	0.329	0.200
11	0.629	0.429	0.771	0.143
12	0.743	0.529	0.429	0.271
13	0.643	0.657	0.486	0.500
14	0.600	0.571	0.586	0.457
15	0.743	0.643	0.600	0.700
16	0.914	0.971	0.700	0.943
mean	0.675	0.539	0.591	0.412

**Table 9:** Confusion Matrix for User S01 KNN.

		Predicted Class						
		Neutral State	Joy	Surprise	Fear	Anger	Sadness	Disgust
True class	Neutral state	10	0	0	0	0	0	0
	Joy	0	10	0	0	1	0	0
	Surprise	2	0	3	1	0	0	4
	Fear	0	0	0	10	0	0	0
	Anger	0	0	0	0	6	4	0
	Sadness	0	0	0	0	7	3	0
	Disgust	3	0	0	4	0	2	1

**Table 10:** Accuracy of Subject-Independent Classification for pairs of emotions.

Emotion Pair	KNN	SVM_Linear	SVM_Cubic	LDA
neutral-joy	0.978	0.963	0.984	0.841
neutral-sadness	0.903	0.919	0.972	0.794
neutral-surprise	0.988	0.916	0.909	0.825
neutral-disgust	0.966	0.953	0,978	0.875
neutral-anger	0.900	0.938	0.938	0.747
neutral-fear	0.847	0.919	0.919	0.816
joy-sadness	0.934	0.931	0.978	0.753
joy-surprise	0.934	0.966	0.934	0.791
joy-disgust	0.963	0.934	0.903	0.844
joy-anger	0.953	0.903	0.891	0.762
joy-fear	0.850	0.953	0.928	0.806
sadness-surprise	0.916	0.938	0.875	0.853
sadness-disgust	0.891	0.928	0.916	0.706
sadness-anger	0.975	0.853	0.900	0.713
sadness-fear	0.828	0.963	0.916	0.787
surprise-disgust	0.781	0.759	0.775	0.753
surprise-anger	0.659	0.756	0.753	0.644
surprise-fear	0.619	0.572	0.441	0.544
disgust-anger	0.859	0.484	0.547	0.503
disgust-fear	0.772	0.744	0.709	0.731
anger-fear	0.775	0.775	0.647	0.678

**Table 11:** Comparison of the Results.

Citation	EMG Channels	No. of Subjects	Results
Zhang, Z., et al. (2022) [37]	2	12	ELM classifier: 60.08% accuracy, SVM: 50% accuracy for positive, negative, and neutral moods.
Kehri, V., et al. (2018) [35]	2 (bipolar)	12	91.66% accuracy for happiness, anger, and disgust emotions.
Jerritta, S., et al. (2014) [22]	2	15	Mean recognition rate of 69.5% for six emotional states.
Barigala, V.K., et al. (2023) [38]	3	30	67.29% to 70.6% accuracy depending on EMG signal location.
Shiva, J., et al. (2021) [39]	2 (bipolar)	32	61.37% accuracy for emotions in the valence dimension using spectral features.
Mithbavkar, S., and Shah, M. (2019) [24]	1	5	NARX neural network: 99.1% maximum accuracy for emotion recognition.
Mithbavkar, S. (2019) [40]	8	3	LSSVM classifier: 69.63% accuracy without head motion and 80.3% with head motion, for nine emotions.
Our	8	16	Subject-dependent: 0.963 for KNN, 0.949 for SVM with linear kernel, 0.946 for SVM with cubic kernel, and 0.938 for LDA. Subject-independent: depending on the tested user and ranged from 48.6% to 91.4% for the KNN classifier, with an average accuracy of 67.5%, for SVM with a linear kernel 53.9%, for SVM with a cubic kernel 59.1%, and the LDA classifier at 41.2%

## Data Availability

EMG Data Recorded During Emotional Expressions. The database has been made available on the website https://github.com/kolodzima/EMG_emotion_data (accessed on 15 July 2024).
